# Iron promotes protein insolubility and aging in *C. elegans*

**DOI:** 10.18632/aging.100689

**Published:** 2014-09-25

**Authors:** Ida M. Klang, Birgit Schilling, Dylan J. Sorensen, Alexandria K. Sahu, Pankaj Kapahi, Julie K. Andersen, Peter Swoboda, David W. Killilea, Bradford W. Gibson, Gordon J. Lithgow

**Affiliations:** ^1^ The Buck Institute for Research on Aging, Novato, CA 94945, USA; ^2^ Karolinska Institute, Department of Biosciences and Nutrition, S-141 83 Huddinge, Sweden; ^3^ Nutrition and Metabolism Center, Children's Hospital Oakland Research Institute, Oakland, CA 94609, USA; ^4^ Department of Pharmaceutical Chemistry, University of California, San Francisco, CA 94143, USA

**Keywords:** Aging, C. elegans, iron, lifespan, metal homeostasis, protein aggregation

## Abstract

Many late-onset proteotoxic diseases are accompanied by a disruption in homeostasis of metals (metallostasis) including iron, copper and zinc. Although aging is the most prominent risk factor for these disorders, the impact of aging on metallostasis and its role in proteotoxic disease remain poorly understood. Moreover, it is not clear whether a loss of metallostasis influences normal aging. We have investigated the role of metallostasis in longevity of *Caenorhabditis elegans*. We found that calcium, copper, iron, and manganese levels increase as a function of age, while potassium and phosphorus levels tend to decrease. Increased dietary iron significantly accelerated the age-related accumulation of insoluble protein, a molecular pathology of aging. Proteomic analysis revealed widespread effects of dietary iron in multiple organelles and tissues. Pharmacological interventions to block accumulation of specific metals attenuated many models of proteotoxicity and extended normal lifespan. Collectively, these results suggest that a loss of metallostasis with aging contributes to age-related protein aggregation.

## INTRODUCTION

Both metal overload and deficiencies can cause metabolic defects, cell cycle arrest and cell death leading to severe pathologies [1-4]. Therefore, tight regulation of metals is required to avoid adverse consequences of either depletion or excess. Cellular metal content is regulated by complex networks of transporter proteins, metal binding proteins and stress response mechanisms [5]. Many age-related chronic disease states result, in part, from a disruption or failure of these regulatory mechanisms [6]. For example, we previously demonstrated that a failure to regulate metal metabolism is a contributing factor in Parkinson's disease [7] where iron regulation plays a critical role in the loss of dopaminergic neurons in the substantia nigra, the brain region primarily affected by the disorder. Iron uptake is known to increase with age in the brain [8] and to be influenced by dietary intake; we showed that increased neonatal iron ingestion results in PD-like neurodegenerative phenotypes late in life [9]. In addition to playing a role in the formation of toxic oligomers of peptides such as β-amyloid and tau, metals can also cause specific metal-induced neuro-degenerative diseases such as Manganism and Friedreich ataxia, which mimic age-related neurodegenerative disorders [10-12]. Although the involvement of some metals in proteotoxic disease have been identified, the influence of the aging process on overall metallostasis has received less attention, preventing integration of all processes that contribute to protein aggregation with age.

*C. elegans* is widely used in studies of metal toxicity and metal homeostasis [13]. Altering the levels of various metals in the growth media causes developmental delays and abnormalities along with diminished fertility. Copper, manganese, iron and zinc have all been shown to reduce *C. elegans* lifespan at various concentrations [14]. Metal exposure can affect worm physiology in unpredictable ways. For example, we previously showed that aluminum can lengthen or shorten lifespan depending on the concentration [15]. Similarly, exposure to low levels of copper decreases the paralysis rate whereas higher concentrations significantly increase the pathology of Aβ (1-42) transgenic worms [16]. Copper exposure induces detrimental effects on *C. elegans* brood size as well as impairing development [17, 18]. Supplementation of manganese can result in accelerated development and increase in total fertility, while reduced body and brood sizes have also been reported [19, 20]. Manganese overload in *C. elegans* has been reported to result in ROS formation and death of dopaminergic neurons [21, 22] whereas it confers stress resistance and increases lifespan in an oxidative stress-sensitive *mev-1* mutant strain at lower concentrations [19]. In addition to a reduction in lifespan, excess iron can also cause a reduction in body size and chemotaxis disruption in *C. elegans* [23, 24]. This defective locomotive behavior observed following iron treatment suggests reductions in synaptic and/or muscle function. Furthermore, endogenous iron levels are increased in *C. elegans* exposed to ROS inducing agents such as paraquat and heat shock [25] and Aβ expression in the worm also results in disruption of iron homeostasis [26]. Interestingly, while reduced levels of the mitochondrial iron transporter mitoferrin leads to abnormal development and reduced fecundity in the worm, it also significantly extends lifespan [27]. Thus, analogous to humans, slight perturbations of many metals in *C. elegans* results in detrimental effects, rendering metallostasis regulation imperative for the health of the organism.

Aging is characterized by a progressive weakening in protein homeostasis (proteostasis). This results in increased protein aggregation, akin to that observed in a range of neurological and other amyloid diseases. Aging of *C. elegans* results in an accumulation of SDS insoluble proteins, a phenomenon delayed by reduced insulin/IGF-1 signaling, which also extends lifespan [28, 29]. We previously reported that knockdown of certain genes involved in this age-related protein insolubility generally promotes longevity, suggesting that this phenomenon is a detrimental feature of the aging process [29]. Moreover, we have discovered that a number of small molecules that extend lifespan also slow age-related protein aggregation [30]. Taken together, these findings suggest that protein aggregation is an inherent part of the aging process and that manipulation of protein aggregation by genetic or pharmacologic means promotes longevity in *C. elegans*.

To investigate the possible link between metals, protein aggregation and aging, we performed a comprehensive analysis of the metallome with age. Results from this analysis demonstrate that aging of *C. elegans* is associated with the accumulation of iron, copper and manganese. We investigated the effects of manipulating iron on aging and age-related neurodegenerative phenotypes and found that dietary supplementation with 15 mM ferric ammonium citrate (FAC) reduces *C. elegans* lifespan in conjunction with increased levels of insoluble proteins. Additionally, iron enhances toxicity in both Aβ and PolyQ-associated models of protein aggregation. This demonstrates that iron has detrimental effects on aging and disease-related protein aggregation. In order to investigate the proteomic changes in *C. elegans* caused by supplemental dietary iron in a non-biased fashion, we employed a global proteomics approach. Using this approach, we identified several hundreds of proteins that become insoluble with increased dietary iron. Conversely, we found that the divalent metal chelator, Calcium Disodium Ethylenediaminetetraacetic acid (CaEDTA) reduces iron and zinc levels in *C. elegans*, is protective in models of proteotoxicity, and extends lifespan. Taken together, our findings suggest that metal accumulation is an inherent part of the aging process and likely undermines protein homeostasis and longevity.

## RESULTS

### Iron and other metals accumulate with age in *C. elegans*

**Figure 1:**
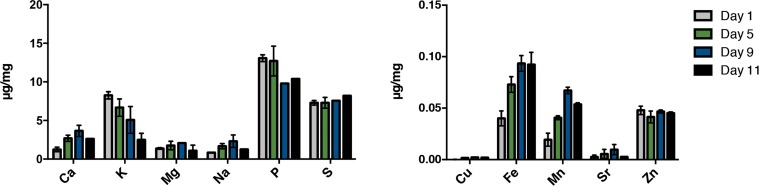
Alteration of the metal profile with age in *C. elegans*. ICPaes analysis of synchronous populations of day 1, day 5, day 9 and day 11 old animals. Metals are grouped by abundance. Bars represent the mean of three biological replicates, each in triplicate. Error bars, SD.

Aging results in losses of various homeostatic mechanisms leading to alterations in the proteomic landscape [31, 32]. We hypothesized that the loss of proteostasis with age may be in part the result of alterations in the levels of metals previously associated with protein aggregation. In order to address this hypothesis, we first tested whether endogenous metal levels change with age. We established a metallomic profile during aging in *C. elegans* by measuring the elemental composition of age-synchronous mass cultures of young hermaphrodites versus older adults. Worms gradually exhibit aging characteristics such as decreased locomotion beginning around day 5 of adulthood at 25°C. The majority of the population lacked spontaneous movement by day 11 of adulthood and over 50 percent of animals were dead at this point. We sampled live animals at day 1, 5, 9 and 11 representing young, young-mid, mid- and late-life animals, avoiding transgenerational contamination by utilizing the temperature conditional sterile strain TJ1060 [*spe-9(hc88) I; fer-15(b26)II*]. 300,000 hermaphrodite worms were maintained for each of the age-synchronous cohorts that were subsequently sampled, snap frozen and prepared for analysis via an inductively coupled plasma-atomic emissions spectrophotometer (ICP-AES; Varian Inc). The abundance of many elements remained at steady levels between day 1 to day 11 of adulthood. However, we found dramatic alterations in levels of many elements known to be associated with human disease. Calcium, iron, copper and manganese levels were found to markedly increase from day one of adulthood in an age-dependent manner while potassium and phosphorus levels decreased (Fig. 1). These changes suggest that aging poses a vulnerability to the metal homeostatic network.

### Increase in dietary iron accelerates protein aggregation in *C. elegans*

**Figure 2:**
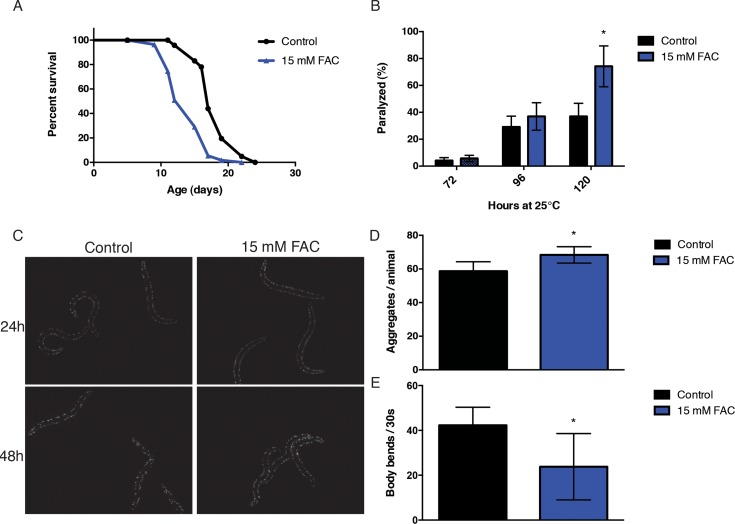
Iron supplementation increases susceptibility to toxicity in models of protein aggregation. (**A**) Kaplan-Meyer curves of wild-type animals exposed to 15 mM FAC from day 1 of adulthood. Iron supplementation significantly decreases lifespan (p<0.0001, Log-rank, Mantel-cox). (**B**) Iron supplementation increases proteotoxicity in an Aβ expressing transgenic strain after 120 hours of exposure (p<0.0001, Student's t-test). (**C**, **D**) Iron supplementation increases the number of polyglutamine inclusions and (E) increases onset of muscle dysfunction in a model of polyglutamine disease after 48 hours of exposure (p<0.0001, p=0.0007 respectively, Student's t-test). Plots are representative of three independent experiments. Error bars, SD.

We noted that iron exhibited a high magnitude of accumulation with aging and we were aware of its role in neurodegenerative-associated protein aggregation. Consequently, we decided to investigate whether an increase in dietary iron could promote aging in *C. elegans*. In agreement with previous reports, we found that supplementation with 15 mM ferric ammonium citrate (FAC) resulted in a modest but robust reduction in mean and maximum lifespan (Fig. 2A). Iron has been shown to affect protein aggregation associated with various neurodegenerative diseases and aging in *C. elegans* has been strongly associated with a loss in proteostasis. In order to investigate whether the effects of dietary iron enrichment on lifespan could be via effects on altered proteostasis, we took advantage of a series of well-characterized *C. elegans* protein aggregation models. We first tested a temperature-conditional strain, CL4176 [*smg-1 (cc546ts); dvIs27(myo-3::Aβ_3–42_ let 39UTR(pAF29))*]. The hall-mark protein associated with Alzheimer's disease (AD) inclusions, Aβ, accumulates in the body wall muscle when this strain is grown at 25°C, leading to paralysis of the animals. We exposed animals to a diet containing 15 mM FAC simultaneously shifting them to 25°C while scoring for the frequency of paralysis each subsequent day. We found that exposure to a diet of 15 mM FAC increased the rate of paralysis in the Aβ transgenic animal (Fig. 2B). Polyglutamine (PolyQ) aggregation is a key pathological hallmark of Huntington's disease (HD) and other related neurodegenerative disorders. We examined PolyQ aggregation in Q40::YFP transgenic animals AM141 *rmIs133[P(unc-54) Q40::YFP)*] upon transient exposure to 15 mM FAC from the young adult stage. We found that iron had a modest but significant effect on the numbers of polyglutamine inclusions in this second protein aggregation model (Fig. 2C, Fig. 2D).

More importantly, an iron-rich environment diet was found to adversely affect functional physiology in this strain as demonstrated by reduction in movement (Fig. 2E). We then tested a strain expressing a metastable mutant perlecan protein; HE250 [*unc-52(e669su250)II*]. Perlecan is the core protein of the mammalian basement membrane heparan sulfate proteoglycan and UNC-52 mutants are often used as indicators of the protein homeostatic network capacity [33]. As with the previous models, worms shifted to the restrictive temperature of 25°C become paralyzed, in this case likely due to a disruption in extracellular matrix fibers. Iron did not, however, exacerbate protein misfolding phenotype in this paradigm (data not shown). This raised the question as to whether specific protein classes are more susceptible to aggregation upon iron supplementation, and if the cellular location plays a significant role in protein vulnerability.

**Figure 3:**
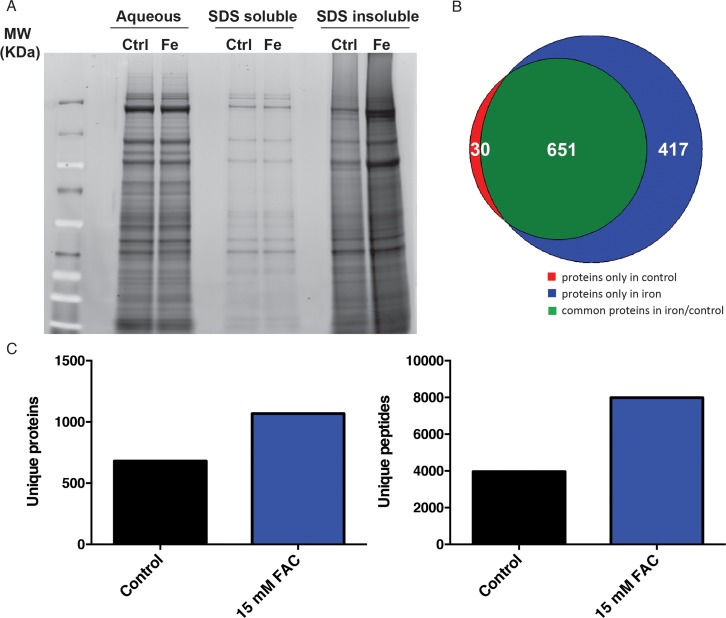
Accumulation of insoluble proteins in iron treated *C. elegans*. (**A**) SDS PAGE gel of the aqueous, SDS-soluble and SDS-insoluble protein fractions of control and iron treated animals. (**B**) Number of insoluble proteins overlapping between control and iron treated animals as identified by Mass Spectroscopy. (**C**) Quantification of proteins and peptides in the SDS-insoluble fraction of control and animals treated with 15mM FAC from day one of adulthood.

Various major disease-specific proteins have been found to become more prone to aggregate or change conformational structure in response to iron supplementation. However, a systematic evaluation of protein aggregation in response to elevated environmental iron exposure to date is lacking. In order to determine proteomic changes in response to iron in a non-biased manner, we undertook a biochemical approach. We and others have shown that a fraction of the *C. elegans* proteome becomes increasingly insoluble with age [28, 29]. The insoluble fraction of the *C. elegans* proteome can be separated with strong detergent buffers such as SDS, where the remaining fraction (the ‘insolublome’) can be re-solubilized by formic acid and visualized on SDS-PAGE gels. We used this approach to identify proteins vulnerable to loss of solubility upon dietary iron supplementation. Consistent with previous findings, we observed that the SDS insoluble protein fraction as assessed by SDS PAGE gel significantly increased with age. Furthermore, 7 day old worms exposed to 15 mM FAC from day 1 of adulthood had a striking increase in insoluble proteins compared with 7 day old controls (Fig. 3A). When comparing age-induced aggregation with aggregation promoted by iron supplementation, we were struck by the apparent similarity in the banding pattern on SDS-PAGE gels. Based on this, we conjectured that elevated dietary iron may result in an acceleration of aging-associated protein insolubility. To further investigate, we proteolytically digested the SDS-insoluble fractions (in an in-solution digestion protocol) and utilized mass spectrometry to first identify and then quantitate proteins within the insoluble fraction in the absence and presence of iron supplementation. Reflecting the SDS PAGE result, we identified 681 proteins for control worms and 1068 proteins for iron-treated worms, with only 30 unique proteins exclusively observed in controls, but 417 unique proteins in iron-treated counterparts (Fig. 3B, Fig. 3C, Table S1 and S2), suggesting that iron potentiates the tendency of numerous proteins to aggregate. Once these sets of aggregation-prone proteins were identified, we looked for common functional or structural features. Using the DAVID v.6.7 software (The Database for Annotation, Visualization and Integrated Discovery) [34], we found a strong similarity in the functional classes of proteins that were identified in iron-supplemented versus control cohorts. Insoluble proteins from iron-supplemented worms were found to be enriched in several functional categories (Kegg pathways) including the ribosome, TCA cycle, oxidative phosphorylation and the proteasome (Table 1A). Interestingly, we observed a strong similarity in the functional classes of aggregation-prone proteins in aged [29] and iron-treated animals, suggesting that iron supplementation and aging have very similar effects on protein insolubility.

**Table 1:** Kegg Pathway enrichment Cutoff: p-value <0.05

All proteins identified in Iron Insolublome (DAVID Ontology), Kegg Pathway enrichment				
Term	Count	%	PValue	Fold Enrichment
**Ribosome**	66	7.0	8.99E-28	3.4
**Citrate cycle (TCA cycle)**	27	2.9	3.93E-12	3.6
**Oxidative phosphorylation**	52	5.5	3.81E-09	2.1
**Valine, leucine and isoleucine degradation**	23	2.4	5.23E-05	2.3
**Proteasome**	20	2.1	3.95E-04	2.2
**Glycolysis / Gluconeogenesis**	18	1.9	0.00126	2.1
**Pyruvate metabolism**	13	1.4	0.00233	2.4
**Aminoacyl-tRNA biosynthesis**	17	1.8	0.00268	2.1
**Propanoate metabolism**	14	1.5	0.00819	2.1
**DNA replication**	15	1.6	0.01094	1.9

We next asked whether a particular cellular compartment or tissue was over-represented in the iron-induced insolublome. We hypothesized that iron, based on its known functions, would induce more specific and localized protein damage compared with the general aging phenotype. However, when we examined expression patterns, we found that iron-induced aggre-gation appears widespread, affecting different tissues and different intracellular compartments, similarly to the aging phenotype (Table S2A). We also used a quantitative label-free mass spectrometry approach (Skyline MS1 Filtering) [35] to determine robust and statistically significant fold changes between control and iron treated worms. Multiple biological, process and technical replicates were used to generate a candidate list (Table 1B). The latter list of 66 changing proteins upon iron treatment was further confirmed in an independent MRM-like quantitative approach called SWATH MS2 [36]. (For all quantitative details see Table S3 and S4). We found that many proteins that become insoluble with age also aggregate in response to iron supplementation, such as several ribosomal proteins, vitellogenins and heat shock protein 60. We also found an interesting seemingly age-independent aggregation by iron supplementation for a smaller set of proteins. For example, iron-sulfur clusters are found in a variety of metalloproteins, but are best known for their role in oxidation-reduction reactions as part of the mitochondrial electron transport chain. We found iron-sulfur proteins to be enriched in the iron-insoluble fraction compared with controls. From the candidate list of robustly increased proteins in iron treated worms we also noted several transthyretin (TTR) proteins, which are known to be involved in human neurodegenerative disease.

### CaEDTA reduces iron levels in *C. elegans*

**Figure 4:**
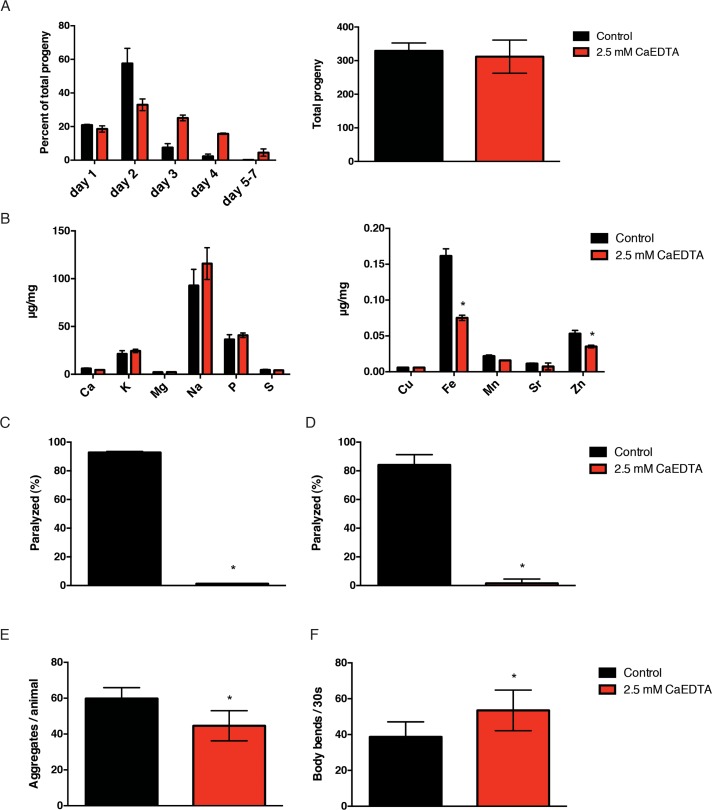
CaEDTA reduces iron and is protective in models of protein aggregation. (**A**) Transient exposure to 2.5mM CaEDTA from the young adulthood stage delays the hermaphrodite reproduction significantly (n=10) but does not result in significant reduction in total progeny (n=10). (**B**) Exposure to CaEDTA from day 1 of adulthood reduces iron (and zinc) levels in 5-day-old animals as measured by ICPaes. Bars represent the mean of three biological replicates. (**C**) CaEDTA delays the paralysis phenotype associated with protein aggregation in an Aβ expressing transgenic and (**D**) perlecan misfolding mutant. (E) CaEDTA exposure prevents the formation of polyglutamine inclusions and (F) reduces the rate of muscle dysfunction in a *C. elegans* model of polyQ aggregation after 48 hours of exposure. Plots are representative of three independent experiments. Error bars, SD (* p<0.0001, Student's t-test).

Based on our data, iron appears to accelerate at least one important feature of normal aging, the accumulation of insoluble protein. We reasoned that if we reduced the impact of metals on insoluble protein formation, we might extend healthspan and possibly lifespan. In order to investigate the role of metal accumulation, we needed an efficient method of reducing the endogenous metal load. Metals are known to play important functions in growth, development and reproduction. After testing various metal chelators, we found that exposure to CaEDTA from the egg stage delayed development and that exposure from the 4^th^ larval stage delayed reproduction (Fig. 4A), suggesting that CaEDTA is readily absorbed and bioavailable in *C. elegans*. In order to test the ability of CaEDTA to reduce metal levels in adult worms, we exposed hermaphrodite worms to CaEDTA continuously from day 1 of adulthood and measured their metal profile by ICPaes 4 days later. We found that exposure to 2.5 mM CaEDTA significantly reduced levels of iron and, to a lesser extent, zinc (Fig. 4B).

### CaEDTA is protective in models of protein aggregation

Since iron exacerbated protein aggregation phenotypes, we speculated that lowering metal levels would ameliorate this effect. We examined the Aβ expressing strain CL4176 for effects of metal chelation via CaEDTA. We s hifted animals to 25°C while simultaneously exposing them to CaEDTA at day 1 of adulthood and subsequently performed daily scoring for paralysis. By day 5 of adulthood, ov er 85% percent of animals in the control group were paralyzed whereas CaEDTA supplementation from time of temperature upshift lead to a nearly complete prevention of the aggregation-induced paralysis (Fig. 4C). When we investigated effects in the perlecan model, we found that exposure to CaEDTA from the time of temperature upshift dramatically suppressed the paralysis phenotype associated with protein misfolding (Fig. 4D). Lastly, we examined formation and toxicity of polyglutamine inclusions in the AM141 strain following CaEDTA treatment. CaEDTA treatment was found to reduce the number of aggregates in this model, as well as delaying onset of muscle dysfunction (Fig. 4E, Fig. 4F). Thus, transient exposure to the metal chelator CaEDTA suppresses pathologies associated with protein mis-folding in several protein aggregation disease models.

**Table 2:** Candidate list of proteins that become insoluble in response to iron-enriched diet and environment. MS1 filtering, protein mean ratio Fe/Control

Gene name	Replicate B2	Replicate B3
**rpt-1**	4.8	10.8
**lec-1**	8.3	4.1
**rps-13**	22.3	3.9
**rps-16**	16.4	4.0
**rps-23**	41.9	9.4
**rps-7**	9.5	3.0
**rps-8**	29.2	3.2
**rps-0**	28.3	4.3
**rla-1**	12.3	4.1
**rpa-2**	5.7	3.8
**rpl-20**	41.5	4.1
**rpl-19**	51.5	2.7
**rpl-6**	6.7	3.1
**nex-1**	23.7	4.4
**nex-3**	10.6	5.1
**atp-2**	33.9	2.3
**F58F12.1**	12.7	2.2
**cgh-1**	5.4	3.0
**cpr-6**	8.2	2.3
**pdi-3**	23.0	2.5
**hsp-60**	14.2	3.5
**C25A8.4**	11.3	5.6
**ucr-1**	23.6	2.2
**dim-1**	10.5	2.4
**lec-5**	39.5	2.8
**gpd-2**	9.0	4.2
**rack-1**	17.5	3.7
**his-11**	6.8	6.0
**ifa-4**	17.4	3.4
**ifb-1**	15.7	3.6
**ifb-2**	27.4	3.0
**klp-17**	11.7	2.2
**flu-2**	12.0	3.0
**lmn-1**	17.5	3.1
**lmp-1**	11.6	2.4
**tomm-20**	10.0	3.2
**mlc-1**	30.9	4.0
**myo-3**	12.6	3.7
**unc-54**	15.0	4.3
**tag-210**	11.3	2.1
**rpn-6.1**	14.6	2.3
**nduf-7**	15.8	2.5
**pgk-1**	6.6	3.1
**R05G6.7**	35.8	4.0
**vha-12**	9.8	3.6
**asp-4**	13.3	3.7
**CELE_D1054.10**	36.6	2.1
**CELE_D1054.11**	65.6	6.2
**dlst-1**	19.5	2.5
**CELE_F13G11.3**	37.7	4.7
**hsp-43**	29.8	3.2
**lec-6**	51.3	8.1
**lfi-1**	6.1	3.3
**perm-4**	23.3	3.6
**rps-24**	17.3	5.1
**stl-1**	15.8	3.6
**ttr-24**	14.3	4.6
**ttr-25**	7.8	2.3
**ttr-41**	17.6	3.2
**ttr-45**	8.0	4.6
**ttr-51**	49.2	4.1
**ucr-2.1**	14.0	4.0
**sip-1**	27.6	3.9
**tct-1**	10.7	3.3
**ttr-2**	28.9	8.2
**mec-7**	7.3	2.7

### CaEDTA extends healthspan and lifespan in *C. elegans*

**Figure 5:**
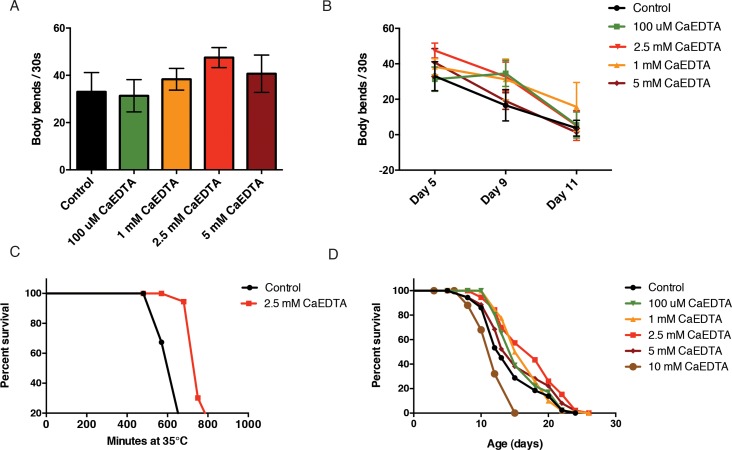
CaEDTA extends healthspan and lifespan in *C. elegans.* (**A**) 2.5 mM CaEDTA exposure from day 1 of adulthood significantly increases motility of 5 day-old animals. (**B**) Transient exposure to CaEDTA from day 1 of adulthood delays age-related decline of movement in wildtype animals in a dose-dependent manner. (**C**) Exposure to 2.5 mM CaEDTA for 24 hours increases thermotolerance in wild-type animals. (**D**) Kaplan-Meyer curve of N2 wildtype worms exposed to 2.5 mM CaEDTA from day 1 of adulthood. Metal chelation significantly increases lifespan (p<0.001, Log-rank, Mantel cox).

We next investigated the effects of CaEDTA on functional aging phenotypes. As a measure of healthspan, we quantified the number of body bends throughout adulthood. We found that CaEDTA slowed spontaneous age-related declines in movement (Fig. 5A, Fig. 5B). We next addressed whether lower metal levels leads to an increase in stress resistance. We exposed day 1 adult animals to CaEDTA and measured survival after an upshift to 35°C. Transient CaEDTA exposure was found to increase the animals' survival upon heat stress (Fig. 5C). Dietary restriction is known to suppress protein aggregation, delay reproduction and increase the lifespan of *C. elegans*. To test whether CaEDTA acts as a dietary restriction mimetic, we measured pharyngeal pumping after treatment. We did not find any significant change in pumping rate upon CaEDTA exposure (Suppl. Fig. 2A), indicating that CaEDTA does not alter food intake. Finally, we asked if reducing the metal load would slow aging by exposing synchronized animals to CaEDTA from day 1 of adulthood and assessing survival. We found that CaEDTA significantly increased the survival of wild-type animals (Fig. 5D).

## DISCUSSION

While several studies have shown correlations between metal imbalance and late-onset disease phenotypes, it is difficult to decipher whether metal accumulation in these conditions are a cause or consequence of the related disorder. Based on results from this current study, we propose that maintenance of physiological metal homeostasis may be causatively associated with healthy aging and longevity in *C. elegans* and that endogenous alterations in the metallome can have consequences for the overall health of the organism.

Several of the age-related metal alterations we observed in our study may directly contribute to known aging phenotypes. For example, an excess of calcium ions in the worm's pharyngeal muscle has been correlated with loss of its mechanical function [37]. Since a balance in calcium levels is crucial for muscle contractility it is possible that the observed accumulation of calcium in aging *C. elegans* contributes to the age-related slowing in body movement and the reduced pharyngeal pumping rate in older animals. Observed decreases in other elements with age in our study may contribute to effects on other age-related physiological dysfunctions.

Lowered potassium levels, for example, can lead to hypokalemia, effecting muscle function. Hypokalemia has serious consequences on human health via its association with a higher risk of atrial fibrillation and cardiac arrest, common heart problems in elderly [38]. Phosphorus is needed for maintenance and repair of all tissues and cells, as well as production of DNA and RNA. It also facilitates use of other minerals and vitamins, including vitamin D, iodine and zinc. Future experiments will be required to learn whether a decrease in these elements have deleterious consequences in *C. elegans*.

Interestingly, many of the metals found to accumulate with age in *C. elegans* are associated with late-onset human neurodegenerative disorders. Numerous publications have described elevated levels of copper, iron and manganese in neurodegenerative patients' brain tissue, cerebrospinal fluid and plasma [39-41]. Our data suggests that these metals tend to increase in older animals. Iron and copper levels undergo progressive changes in conjunction with the advance of neurodegeneration, although the mechanisms involved are unclear, this is consistent with metal accumulation causing a decline in brain function [42]. Iron displayed one of the most dramatic endogenous increases with age in our study. We observed that dietary iron supplementation in *C. elegans* results in increased protein aggregation in various models of proteotoxic diseases. In human aging, iron accumulates in brain gray matter regions and may contribute to the risk of developing AD. *In vitro* studies have shown that iron can adhere to Aβ, accelerating fibril elongation and promoting aggregation [43, 44]. Consistent with these reports, we found that supplemental iron can worsen the pathology in a *C. elegans* model of amyloid-β aggregation. HD is associated with increased iron in basal ganglia [45] and we found that supplemental iron exaggerate polyglutamine inclusions as well as impaired the motility of a *C. elegans* PolyQ transgenic model.

Similarly to normal aging in *C. elegans*, we determined that iron supplementation resulted in a large increase in the number of SDS-insoluble proteins. Furthermore, there is a striking similarity between the functional classes of proteins affected by both aging and iron. As with the aging-induced data sets, we found a significant enrichment in various biological processes including metabolic processes, biogenesis, embryonic development and apoptosis. Overall, this data suggests that iron supplementation in many ways mimics the natural aging phenotype, raising the possibility that iron could contribute to the aging process itself.

Metal ions are essential for the function of over one third of all proteins and are involved in a number of key biological processes including respiration. When analyzing the iron-induced insoluble fraction, we identified a significant enrichment in proteins localized to the mitochondria, including numerous proteins involved in the electron transport chain (Suppl. Fig. 1). Heme and iron-sulfur (Fe-S) clusters are essential for the function of ROS-producing enzymes such as NADPH and oxidases involved in mitochondrial function. Consequently, redox-active pools of endogenous iron are found at high levels in the cytosol and mitochondrial matrix. This so-called “free iron” can catalyze free radica l formation and is thus often suggested a potential perpetrator of pathologies linked to ROS accumulation, including those affecting mitochondrial activity [46]. Furthermore, oxidative stress can cause the Fe-S group in the iron response element binding protein 1 (IRP-1) to collapse, which triggers IRP-1 to stabilize production of transferrin receptor, bringing more iron into the cell, while reducing ferritin levels, which normally stores iron in a form where it cannot participate in redox reactions [47]. Thus, iron supplementation may result in aggregation of iron-sulfur proteins that can profoundly affect mitochondrial function, potentially causing a negative forward-feeding cycle, resulting in further iron accumulation. An iron rich diet had effects on extracellular proteins as well. Several age-related amyloid disorders are characterized by the extracellular deposition of fibrils and aggregates of TTR, which can be exaggerated by protein oxidation [48]. Interestingly, we found that iron treatment promoted insolubility of several TTR proteins in *C. elegans*.

Iron is known to contribute to ROS production, a hallmark trait of the aging process. Both iron and copper are known to bind to amyloid precursor protein in and promote formation of ROS in neuronal cells [49, 50]. Consistent with these reports, Valentini et al previously reported that the iron chelator deferoxamine promotes resistance to oxidative stress in *C. elegans* [24]. However, they found no beneficial effects on lifespan at the concentrations used. In agreement with this, we found no effects on lifespan when exposing worms to various concentrations of deferoxamine, up to the highest soluble dose, 1 mM (data not shown). In contrast, CaEDTA exposure significantly increased healthspan and lifespan in a concentration-dependent manner, independently of the well-studied lifespan modulator DAF-16 (Suppl. Fig 2B). Although deferoxamine has been suggested to lower iron levels in *C. elegans*, actual uptake and level of biological iron reduction has not been demonstrated. In our study, we have shown a significant depletion in endogenous iron in the presence of CaEDTA. Moreover, while CaEDTA exposure from egg or larval stages delayed development, deferoxamine appeared to have no effect on development nor reproduction in our hands. This could suggest that the drug is not as bioavailable to *C. elegans*. Alternatively, a more broad-based chelator, CaEDTA might be promoting homeostasis of a wider spectrum of metals.

It is often suggested that metal chelation could have beneficial effects on aging, by reducing the likelihood of oxygen radical formation. However, a number of studies in recent years imply that oxidative damage does not cause aging in *C. elegans* [51-53]. Another potential explanation by which metal chelation could result in longevity is by proteostatic stabilization. Deficiencies in proteostasis may lead to cell dysfunction and disease. Our studies suggest that lowering metal levels via chelation can slow protein aggregation commonly associated with the aging process.

Nutritional effects on aging and age-related disease are dramatic such as those observed in dietary restricted mice [54, 55]. The effects of dietary fat, carbohydrates and calories on organismal aging are all well studied. We suggest that many other aspects of nutrition, including dietary metal composition, may play a vital role in the aging process, including influencing protein homeostasis. Maintenance of the metallome may therefore constitute a novel therapeutic target for aging as well as age-related diseases.

## METHODS

### Caenorhabditis elegans strains

The TJ1060 strain [*spe-9(hc88)I; fer-15(b26)II*] strain was a gift from Thomas E. Johnson (Univ. of Colorado, Boulder). The following worm strains were obtained from the *Caenorhabditis* Genetics Center (CGC, University of Minnesota) and cultured using standard conditions: Bristol N2 (wild-type), CB4037 [*glp-1(e2141)*], CF1038 [*daf-16(mu86)*]. For paralysis and motility experiments, the following strains were used: CL4176 [*smg-1 (cc546ts); dvIs27(myo-3::Aβ_3–42_ let 39UTR(pAF29))*]; *pRF4*], HE250 [*unc-52(e669su250)II*], AM141 [*rmIs133(P(unc-54) Q40::YFP)*].

### Elemental analysis

Synchronous populations of 300,000 worms were grown in mass cultures on enriched peptone plates with NA22 as the food source [56]. 300 μL of worm pellet per sample was collected on day 1, 5, 9 and 11, respectively, with isotonic buffer (HEPES, choline chloride Omnitrace metal free water), dead animals were omitted. Worms exposed to 2.5 mM CaEDTA were collected at day 5 of adulthood, after continuous exposure from day 1 of adulthood. The worms were pelleted by centrifugation and washed in isotonic buffer three times over a total of 30 minutes to clear gut content. The worm pellets were dried for 48 hours at 60°C. Samples were digested in 250 μL of 70% HNO_3_. Samples were diluted to a final volume of 3.125 ml at 5% HNO_3_ for ICPaes analysis with a Vista Pro inductively coupled plasma-atomic emissions spectrophotometer (ICP-AES; Varian Inc).

### Adult lifespan assays

Compound preparation: Solutions of CaEDTA and FAC were prepared at 75 mM and 450 mM concentrations respectively. Stocks were sterile filtered and 100 μL of solution spotted onto 3mL NGM/OP50 *E. coli* plates at a final concentration of 2.5 and 15 mM respectively. Control plates were spotted with 100 μL water (CaEDTA) and 9% ammonium citrate (FAC). Plates were dried at room temperature for 24 hours and stored and 4°C for no longer than one week.

60 or more synchronous hermaphrodite worms were grown at 20°C on standard NGM/OP50 plates and transferred to CaEDTA or FAC on day 1 of adulthood. The worms were transferred every day for one week and every third day subsequently while scoring for survival. Animals that crawled off the plates or died due to internal hatching were censored. All experiments were done in the absence of FUdR.

### Aggregation-associated paralysis assays

CL4176: Synchronous populations were grown at 15°C until the animals were transferred onto drugs (L4 stage FAC exposure, day 1 adult CaEDTA exposure) and simultaneously shifted to 25°C to allow for Aβ expression. The number of paralyzed animals was scored every day post temperature upshift.

HE250: Synchronous populations were grown at 15°C until the young adult stage at which point the animals were transferred onto compounds and simultaneously shifted to the permissive temperature of 25°C. Paralysis was scored 48 hours post temperature upshift.

### Microscopy and quantification

Animals were paralyzed in 500 uM levamisole and mounted on 2 mM agar pads with glass coverslips. YFP expression was analyzed using an Olympus BX51 upright microscope. Quantification of inclusions was done using ImageJ^TM^.

### Motility assays

Movement was measured by placing individual day 2 adult animals in 10uL S-basal and allowed to recover for 20 seconds. The number of body bends was then counted for 30 seconds.

### Insoluble protein extraction

TJ1060 [*spe-9(hc88)I; fer-15(b26)II*] temperature sensitive mutants were grown in synchronous mass cultures and exposed to compound on day one of adulthood. Samples of approximately 300 μL were collected and the worms were washed several times with S-basal. Samples were snap frozen, thawed on ice and resuspended in aqueous lysis buffer (20 mM Tris base, 100 mM NaCl, 1 mM MgCl_2_, pH=7.4) with protease inhibitor cocktail COMPLETE™ (Roche Diagnostics, Mannheim, Germany). The worms were sonicated on ice and then briefly centrifuged at 3000xg to remove remaining carcasses. All samples were normalized for total protein concentration as assessed by BCA assay. Samples were centrifuged at 16,000xg in 4°C and washed in lysis buffer. This step was repeated three times. The pellet was resuspended and washed in lysis buffer containing 1% SDS and centrifuged at 16,000xg. This step was repeated three times to retain the SDS insoluble fraction. The remaining pellet was then treated with 100 μL of 70% formic acid for one hour to dissolve the SDS insoluble fraction. The acidic fractions were dried for 2 hours in a Speed-Vac at 45°C.

### Mass spectrometry and protein quantification

The formic acid re-suspended insolublome pellets obtained from 7 day old worms exposed to 15 mM FAC and 7 day old controls, respectively, were denatured in a final solution of 6 M urea, and 100 mM Tris and further processed for proteolytic digestion. The protein mixture was reduced with 20 mM DTT (37°C for 1 h), and subsequently alkylated with 40 mM iodoacetamide (30 min at RT in the dark). Samples were diluted 10-fold with 100 mM Tris pH 8.0 and incubated overnight at 37°C with sequencing grade trypsin (Promega, Madison WI) added at a 1:50 enzyme:substrate ratio (wt/wt). Samples were then acidified with formic acid and desalted using HLB Oasis SPE cartridges (Waters, Milford MA). Proteolytic peptides were eluted, concentrated to near dryness by vacuum centrifugation, re-suspended and further desalted (C-18 zip-tips) for mass spectrometric analysis. Iron treated samples and control samples of 7 day old worms were each prepared in 3 separate biological replicates, and each in two independent process (sample preparation/workflow) replicates. Technical duplicates (MS injection duplicates) were acquired to assess technical variability. All samples were analyzed by reverse-phase HPLC-ESI-MS/MS using an Eksigent UltraPlus nano-LC 2D HPLC system (Dublin, CA) connected to a quadrupole time-of-flight TripleTOF 5600 mass spectrometer (AB SCIEX). Typically, mass resolution for MS1 scans and corresponding precursor ions was ~35,000 while resolution for MS2 scans and resulting fragment ions was ~15,000 (‘high sensitivity’ product ion scan mode). Specifically, samples were acquired by reverse-phase HPLC-ESI-MS/MS using an Eksigent Ultra Plus nano-LC 2D HPLC system (Dublin, CA) which was directly connected to a quadrupole time-of-flight (QqTOF) TripleTOF 5600 mass spectrometer (AB SCIEX, Concord, CAN). The auto sampler was operated in μl-pickup injection mode filling a 3 μl loop with 3 μl analyte per injection. Briefly, after injection, peptide mixtures were transferred onto the analytical C18-nanocapillary HPLC column (C18 Acclaim PepMap100, 75 μm I.D. × 15 cm, 3 μm particle size, 100 Å pore size, Dionex, Sunnyvale, CA) and eluted at a flow rate of 300 nL/min using the following gradient: at 5% solvent B in A (from 0-13 min), 5-35% solvent B in A (from 13-58 min), 35-80% solvent B in A (from 58-63 min), at 80% solvent B in A (from 63-66 min), with a total runtime of 90 min including mobile phase equilibration. Solvents were prepared as follows, mobile phase A: 2% acetonitrile/98% of 0.1% formic acid (v/v) in water, and mobile phase B: 98% acetonitrile/2% of 0.1% formic acid (v/v) in water. For selected samples, additional data sets were recorded in data-independent mode (DIA) using SWATH MS2 acquisitions for quantitative analysis. In the SWATH-MS2 acquisition, a Q1 window of 25 *m/z* was selected to cover the mass range of *m/z* 400-1000 in 24 segments (24 × 100 msec), yielding a cycle time of 3.25 sec, which includes one 250 msec MS1 scan. Data acquisition was performed in data dependent mode (DDA) on the TripleTOF 5600 to obtain MS/MS spectra for the 30 most abundant precursor ions (approx. 50 msec per MS/MS) following each survey MS1 scan (250 msec). Each sample was analyzed in 3 biological and 3 technical injection/MS replicates. For protein identification all data were searched using Protein Pilot v. 4.5 beta [57] using a false discovery rate (FDR) of 1%. For MS instrumentation details and bioinformatics database search engine specifics see Procedure S1. MS1 chromatogram based quantification was performed in Skyline 2.5 an open source software project (http://proteome.gs.washington.edu/software/skyline) as described recently in detail by Schilling et al. [35]. A candidate protein list showing a robust increase in iron-treated versus control worms was confirmed quantifying additional, MRM-like data-independent SWATH MS2 acquisitions. Mass spectrometric raw data can be accessed at ftp://ftp.buckinstitute.org:225/Iron_Insoluble_Proteins/. The processed MS data is provided in Table S1-S4 to show comprehensive lists of peptides that were identified with all their mass spectrometric information, as well as detailed MS1 Filtering and SWATH quantification results. Annotated MS/MS spectral libraries can be accessed at https://daily.panoramaweb.org/labkey/project/Gibson/Lithgow_Iron/begin.view?

### Thermotolerance assays

Briefly, animals were grown at 20°C and transferred to drug plates on day one of adulthood. After 10-14 hours of exposure, the temperature was raised to 35°C and animals were scored for survival beginning after 5 hours and every 1.5 hour thereafter until all animals were dead. Data analyses for lifespan and thermotolerance assays were carried out using Prism™ (GraphPadSoftware Inc., SanDiego,USA).

## SUPPLEMENTAL INFORMATION FIGURES AND TABLES




