# Supplementary material for: Iron promotes protein insolubility and aging in C. elegans
Source: Aging (Albany NY). 2014 Sep 25;6(11):975–88. doi: 10.18632/aging.100689 (PMC4276790; doi:10.18632/aging.100689)
Supplement: Supplementary file 1 [file aging-06-975-s001.pdf]

SUPPLEMENTAL INFORMATION

SUPPLEMENTAL PROCEDURES

Procedure S1. Materials. HPLC solvents including acetonitrile and water were obtained from Burdick & Jackson (Muskegon, MI). Reagents for protein chemistry including iodoacetamide, dithiothreitol (DTT), ammonium bicarbonate and formic acid were purchased from Sigma Aldrich (St. Louis, MO). HLB Oasis SPE cartridges were purchased from Waters (Milford, MA), and proteomics grade trypsin was from Promega (Madison WI). Trypsin-predigested beta-galactosidase (a mass spectrometric quality control standard) was purchased from AB SCIEX (Foster City, CA).

Mass Spectrometry. All samples were analyzed by reverse-phase HPLC-ESI-MS/MS using an Eksigent Ultra Plus nano-LC 2D HPLC system (Dublin, CA) connected to a quadrupole time-of-flight TripleTOF 5600 mass spectrometer (AB SCIEX). Typically, mass resolution for MS1 scans and corresponding precursor ions was ~35,000 while resolution for MS2 scans and resulting fragment ions was ~15,000 ('high sensitivity' product ion scan mode). Data acquisition was performed in data dependent mode (DDA) on the TripleTOF 5600 to obtain MS/MS spectra for the 30 most abundant precursor ions (approx. 50 msec per MS/MS) following each survey MS1 scan (250 msec). Each sample was analyzed in 3 technical injection/MS replicates. Specifically, samples were acquired by reverse-phase HPLC-ESI-MS/MS using an Eksigent Ultra Plus nano-LC 2D HPLC system (Dublin, CA) which was directly connected to a quadrupole time-of-flight (QqTOF) TripleTOF 5600 mass spectrometer (AB SCIEX, Concord, CAN). The autosampler was operated in µl-pickup injection mode filling a 3 µl loop with 3 µl analyte per injection. Briefly, after injection, peptide mixtures were transferred onto the analytical C18-nanocapillary HPLC column (C18 Acclaim PepMap100, 75 µm I.D. x 15 cm, 3 µm particle size, 100 Å pore size, Dionex, Sunnyvale, CA) and eluted at a flow rate of 300 nL/min using the following gradient: at 5% solvent B in A (from 0-13 min), 5-35% solvent B in A (from 13-58 min), 35-80% solvent B in A (from 58-63 min), at 80% solvent B in A (from 63-66 min), with a total runtime of 90 min including mobile phase equilibration. Solvents were prepared as follows, mobile phase A: 2% acetonitrile/98% of 0.1% formic acid (v/v) in water, and mobile phase B: 98% acetonitrile/2% of 0.1% formic acid (v/v) in water.

For selected samples, additional data sets were recorded in data-independent mode (DIA) using SWATH MS2 acquisitions for quantitative analysis. In the SWATH-MS2 acquisition, a Q1 window of 25 *m/z* was selected to cover the mass range of *m/z* 400-1000 in 24 segments (24 x 100 msec), yielding a cycle time of 3.25 sec, which includes one 250 msec MS1 scan.

Bioinformatic database searches. Mass spectrometric data was searched using the database search engine ProteinPilot (Shilov, 2007 #121) (AB SCIEX Beta 4.5, revision 1656) with the Paragon algorithm (4.5.0.0, 1654). The search parameters were set as follows: trypsin digestion, cysteine alkylation set to iodoacetamide, urea denaturation, and species *C. elegans*. Trypsin specificity was assumed as C-terminal cleavage at lysine and arginine. Processing parameters were set to "Biological modification" and a thorough ID search effort was used. During the search, Protein Pilot performs an automatic mass recalibration of the data sets based on highly confident peptide spectra. All data files were searched using the SwissProt 2012_07 database) with a total of 6716 *C. elegans* protein sequences. For Protein Pilot Searches, to assess and restrict rates of false positive peptide/protein identifications, we used the Proteomics System Performance Evaluation Pipeline (PSPEP) tool available in ProteinPilot 4.5 beta, respectively. This tool automatically creates a concatenated forward and reverse decoy database, and provides an Excel output of the experimentally determined false discovery rate at the spectral, peptide and protein levels with standard statistical errors. The discriminating variable for the Paragon Algorithm is the peptide confidence value, which is a 0-99 scaled real number (Shilov, 2007 #121). For database searches, a cut-off peptide confidence value of 99 was chosen with the following justification. For searching the databases the Protein Pilot false discovery rate (FDR) analysis tool (PSPEP) algorithm (Shilov, 2007 #121) provided a global FDR of 1% and a local FDR at 1% in all cases.

Quantitative Skyline MS1 Filtering Analysis. MS1 chromatogram based quantification was performed in Skyline 2.5 an open source software project (http://proteome.gs.washington.edu/software/skyline) as recently described recently in detail by Schilling et al (Schilling, 2012 #161). Briefly, first, comprehensive spectral libraries were generated in Skyline from database searches of the raw data files prior to MS1 Filtering. Second, all raw files acquired in data dependent acquisition mode (DDA), are directly imported into Skyline 2.5 and MS1 precursor ions are extracted for all peptides present in the MS/MS spectral libraries (MS1 resolution setting in Skyline for precursor ion extraction is set at 10,000). Quantitative analysis is based on extracted ion chromatograms

(XICs) and resulting precursor ion peak areas for M, M+1, and M+2; final quantitative comparisons are typically based on only the highest ranked precursor ion. Precursor peak areas were exported from Skyline into excel sheets using the recently developed statistical program 'MS1Probe' (D'Souza, 2013), a Python-coded bioinformatics program that interfaces with Skyline 'external tools' (http://www.gibsonproteomics.org/resources/MS1Probe) to calculate ratios per peptide between different experimental conditions (i.e., control vs. iron treated worms). Significance was assessed using two-tailed Student's t-test requiring ρ values of $\rho < 0.05$.

Raw Data accession and Panorama Spectral Libraries. The raw and processed data (i.e., Skyline files) associated with this manuscript may be downloaded from the Buck Institute ftp site at ftp://ftp.buckinstitute.org:225/Iron_Insoluble_Proteins/. Details for peptide quantification using MS1 Filtering, including peptide peak areas for all MS replicates are provided as excel files as well as summarized in Supplemental Tables S3-S4. A spectral library with MS/MS spectra for all peptides/proteins used for MS1 Filtering and subsequent quantification was transferred and published to the interactive, web-based data sharing Panorama server (Sharma et al, 2012). The spectral viewer can be assessed at https://daily.panoramaweb.org/labkey/project/Gibson/Lithgow_Iron/begin.view?

Funtional Analysis - Protein Ontology. The web-based program DAVID v.6.7 (The Database for Annotation, Visualization and Integrated Discovery), (Huang da, 2009 #120) was used for functional analysis and protein ontology analysis. Analysis was performed for proteins identified in the Insolubelome by mass spectrometry that were significantly and robustly increased in their relative abundance comparing 'control worms' to 'iron-treated worms'.

SUPPLEMENTAL REFERENCES

I. Shilov V et al. The Paragon Algorithm, a next generation search engine that uses sequence temperature values and feature probabilities to identify peptides from tandem mass spectra. Molecular & Cellular Proteomics. 2007; MCP 6, 1638.

2. Schilling B et al., Platform-independent and label-free quantitation of proteomic data using MS1 extracted ion chromatograms in skyline: application to protein acetylation and phosphorylation. Molecular & cellular proteomics. 2012 (May); MCP 11, 202.

3. D'Souz AK a et al. MS1 Probe – Implementation of a Statistical Tool for MS1-based Quantitation in Skyline for High Throughput Quantitative Analysis. Proceedings of the 61st Annual ASMS Conference on Mass Spectrometry & Allied Topics, Minneapolis, MN, 2013; June 8-13.

4. Sharma V et al., Panorama: A Private Repository of Targeted Proteomics Assays for Skyline. Proceedings of the 60th Annual ASMS Conference on Mass Spectrometry & Allied Topics, Vancouver, CAN; 2012; May 20-24.

5. Huang da W, Sherman BT, Lempick RA. Systematic and integrative analysis of large gene lists using DAVID bioinformatics resources. Nature protocols. 2009; 4, 44.

SUPPLEMENTAL TABLES

Please browse full text version of this manuscript to see the data of Supplemental Tables.

SUPPLEMENTAL FIGURES

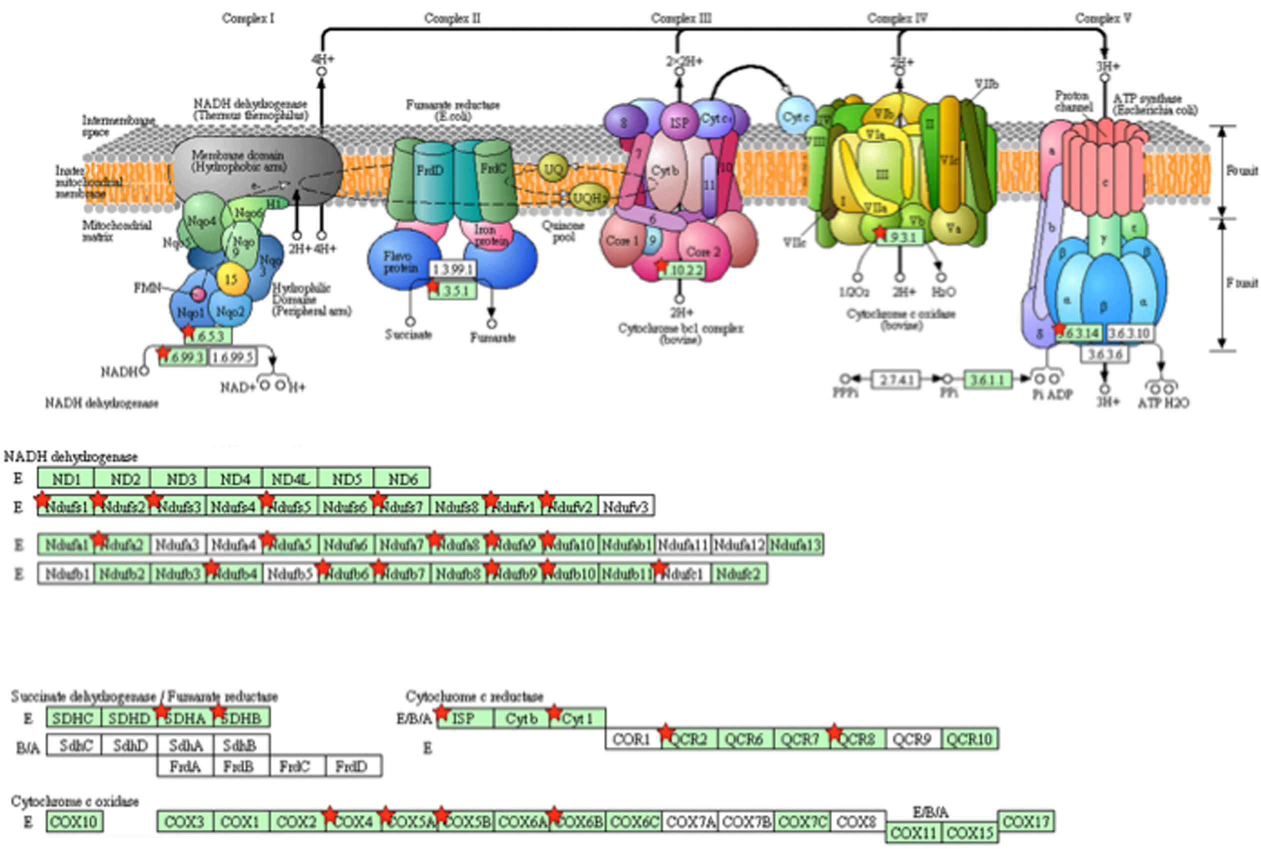

Supplemental Figure 1. An iron rich diet causes insolubility of proteins involved in oxidative phosphorylation. Observed proteins in iron insolublome marked with asterisk. GO Ontology DAVID.

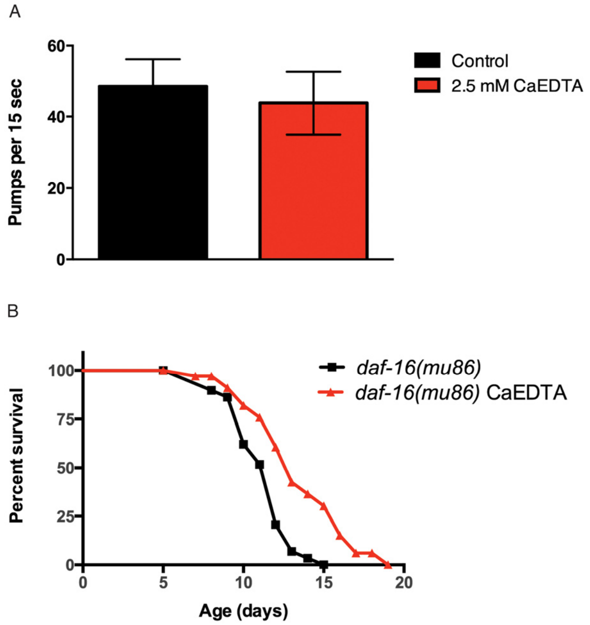

Supplemental Figure 2. (**A**) Exposure to 2.5 mM CaEDTA for 24 hours does not alter feeding rates. (**B**) 2.5mM CaEDTA Kaplan-Meyer survival curve of CF1038 [daf-16(mu86)I] (p<0.001, Log-rank, Mantel cox). Curves are representative of three independent experiments. In each survival experiment, n<60 for each cohort.
